# Seminal Plasma Induces Overexpression of Genes Associated with Embryo Development and Implantation in Day-6 Porcine Blastocysts

**DOI:** 10.3390/ijms21103662

**Published:** 2020-05-22

**Authors:** Cristina A. Martinez, Josep M. Cambra, Maria A. Gil, Inmaculada Parrilla, Manuel Alvarez-Rodriguez, Heriberto Rodriguez-Martinez, Cristina Cuello, Emilio A. Martinez

**Affiliations:** 1 Department of Biomedical & Clinical Sciences (BKV), BKH/Obstetrics & Gynaecology, Faculty of Medicine and Health Sciences, Linköping University, SE-58185 Linköping, Sweden; cristina.martinez-serrano@liu.se (C.A.M.); manuel.alvarez-rodriguez@liu.se (M.A.-R.); heriberto.rodriguez-martinez@liu.se (H.R.-M.); 2 Department of Medicine and Animal Surgery, Faculty of Veterinary Medicine, International Excellence Campus for Higher Education and Research “Campus Mare Nostrum”, University of Murcia, 30100 Murcia, Spain; josepmiquel.cambra@um.es (J.M.C.); mariagil@um.es (M.A.G.); parrilla@um.es (I.P.); emilio@um.es (E.A.M.); 3 Institute for Biomedical Research of Murcia (IMIB-Arrixaca), Campus de Ciencias de la Salud, Carretera Buenavista s/n, 30120 El Palmar, Murcia, Spain

**Keywords:** seminal plasma, embryo, transcriptome, preimplantation, embryo transfer, pig

## Abstract

The infusion of boar seminal plasma (SP) before artificial insemination (AI) positively alters the expression of endometrial genes and pathways involved in embryo development. This study aimed to determine which transcriptome changes occur in preimplantation embryos in response to SP infusions during estrus. Postweaning estrus sows received 40-mL intrauterine infusions of either SP (*N* = 6) or BTS extender (control group; *N* = 6) 30 min before each of two post-cervical AIs. On Day 6, embryos were surgically collected and analyzed for differential gene expression. Microarray analysis of embryos revealed 210 annotated genes, differentially expressed (*p*-value < 0.05 and fold change </> 2) in SP-blastocysts, compared to controls. Most of these genes were associated with biological, cellular, metabolic and developmental processes. The pathways enriched among the upregulated genes related to signal transduction, cellular processes and the endocrine system. Among altered genes involved in these pathways, the SP-group showed a conspicuous overexpression of *ApoA-I*, *CDK1*, *MAPK1*, *SMAD2*, *PRKAA1* and *RICTOR*, with reported key roles in embryo development, implantation, or progression of pregnancy. In conclusion, the results demonstrate that SP infusions prior to AI upregulates the expression of embryo development related genes in Day 6 pig embryos.

## 1. Introduction

In addition to the well-known actions of seminal plasma (SP) on sperm transport and survival, its important roles in the female’ reproductive system and its influence on multiple reproductive processes had been studied [1,2,3]. 

In the pig, the SP shortens the LH peak-to-ovulation interval [4], advancing ovulation [5], and supports luteal development and, consequently, the synthesis of progesterone [6]. In addition, the SP induces endometrial changes in the expression of numerous genes related to maternal immunity during the peri-ovulation period [7,8]. In rodents, SP infusions at estrus support pre-implantation embryonic development and implantation [9], by likely modifying the release of cytokines that support embryonic development, and by inducing the expression of factors associated with angiogenesis and chorionic attachment [10]. 

For all the above-mentioned reasons, a reproductive technology that could benefit from the effects of SP is somatic cell nuclear transfer (SCNT). Both SP-enrichment of media for culture of porcine SCNT embryos and/or SP intra-uterine infusion of recipients prior to intra-oviductal transfer of SCNT embryos appear attractive options since SP-factors can enhance their pre- and post-implantation developmental outcome. Moreover, SP-factors can improve the cytological and molecular qualities of SCNT-embryos to levels found in other livestock species; as the relatively low capability of donor cell nuclear genome to be epigenetically reprogrammed, the impairments in intergenomic communication between nuclear and mitochondrial DNA fractions in SCNT-derived oocytes and resultant embryos, and the increased incidence of programmed cell death in the ex vivo expanded nuclear donor cells, and the corresponding cloned embryos [11,12,13,14,15]. 

In addition, SP could be of great importance for embryo transfer (ET) technology. For instance, in rodents, ET protocols frequently include SP infusions in the recipient females during estrus [16,17]. Estrus SP-infusions seem to promote embryo growth, survival and implantation [17], suggesting a long-lasting effect. In pigs, SP infusions during estrus influenced the uterine environment during at least the first nine days of pregnancy, with changes of specific endometrial cytokines known to promote embryo development and implantation [18]. Our own findings revealed that SP infusions during estrus not only advance embryo development in pigs, but also change the global endometrial transcriptome at Day 6 of pregnancy [19]. This particular study showed >1600 endometrial transcripts differentially expressed in SP-treated endometrium compared to controls, depicting overrepresentation of genes and pathways potentially involved in embryo development and implantation, such as immune response, cytokine signaling, cell cycle, cell adhesion and hormone response [19]. These results re-confirmed the long-lasting effects of porcine SP estrus-infusions throughout the preimplantation period, suggesting SP-use has a potential application value in ET technology, optimizing ET procedures in both donor and recipient females. However, to the best of our knowledge, molecular studies regarding the direct effects of pig SP on preimplantation embryos are lacking. Therefore, this work aimed to determine the changes in the global transcriptome of Day 6 in vivo derived pig blastocysts in response to SP infusions during estrus.

## 2. Results

### 2.1. Transcriptional Profile of Embryos

**Figure 1:**
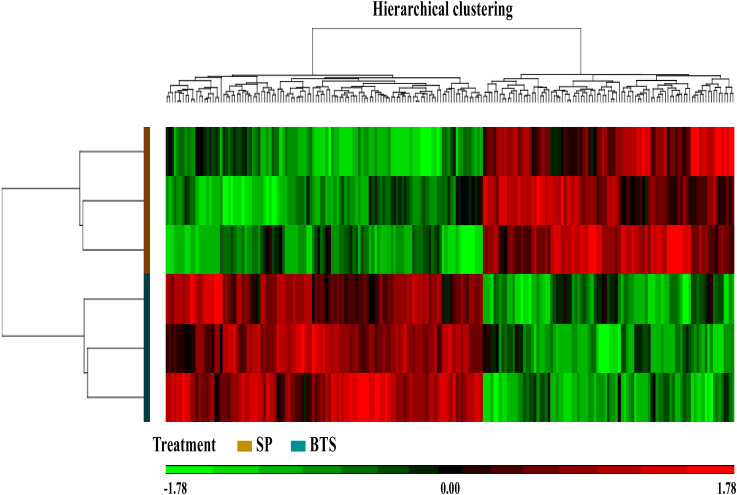
Hierarchical clustering analysis comparing differentially expressed genes for “seminal plasma (SP) vs. BTS-embryos”. Rows and columns represent samples and genes, respectively. Colors correspond to the expression levels of the detected genes upregulated (red) and downregulated (green). Each vertical line represents single gene and each row represents single sample (SPS or BTS). Upper branches indicate relationships among different genes and left branches indicate differences among samples.

We analyzed the transcriptome changes of in vivo-derived blastocysts in response to SP- or BTS (Beltsville thawing solution; [20]) extender (control)-infusions, before each of two consecutive AIs. Following statistical analysis, we identified 311 transcripts that were differentially expressed in SP-embryos relative to BTS-embryos. Among these transcripts, 210 were annotated as known genes (93 upregulated and 117 downregulated; Table S1), with about 30% of transcripts unable to be annotated. Principal component analysis (PCA) of all expressed genes separated SP from BTS samples. The first three PCA-axes accounted for 70.9% of the variability, of which the first axis accounted for 29.7%. By hierarchical clustering analysis, these genes exhibited striking segregation between SP and BTS groups (Figure 1).

### 2.2. Gene Ontology and Pathway Enrichment Analysis of Differentially Expressed Genes (DEGs)

**Figure 2:**
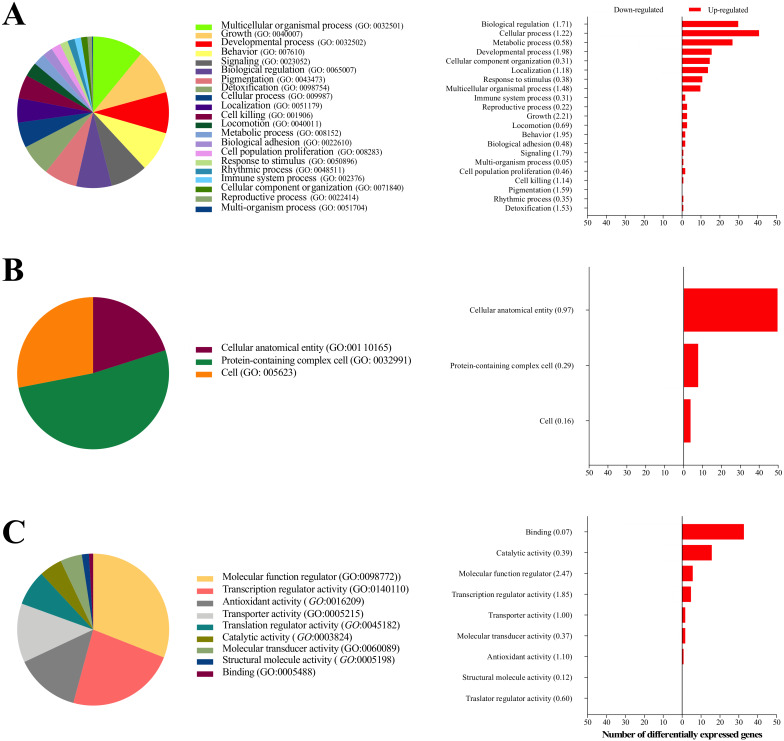
Distribution of differentially expressed genes in embryos derived from seminal plasma-infused sows within biological processes (**A**) cellular components (**B**) and molecular functions (**C**). The distribution of upregulated and downregulated genes within each of biological process, cellular component and molecular function is illustrated in the right-hand side figures (numbers within parentheses are enrichment scores).

Using the Kyoto Encyclopedia of Genes and Genomes (KEGG) database, all DEGs were classified into three main GO categories: biological processes, cellular components and molecular functions (Figure 2).

For biological processes, most of the DEGs were associated with biological, cellular, metabolic and developmental processes (Figure 2A). For cellular components, cell anatomical entity and cell protein-containing complex were the most highly represented categories (Figure 2B). For molecular functions, genes involved in molecular function regulator and transcription regulator activity were most abundant (Figure 2C). In general, within each biological function, similar numbers of upregulated and downregulated genes were observed.

For each dataset, three gene lists, namely: all-DEG, up-DEG and down-DEG containing all genes, up-regulated genes and down-regulated genes, respectively (Table S1), were analyzed to detect significant KEGG pathways. We identified 10 pathways enriched in all-DEG list, 13 pathways enriched in the up-DEG list and three pathways enriched in the down-DEG list. This resulted in a collection of 15 pathways particularly affected by SP infusions (Table 1), of which nine pathways were enriched in both the up-DEG and the all-DEG lists. 

**Figure 3:**
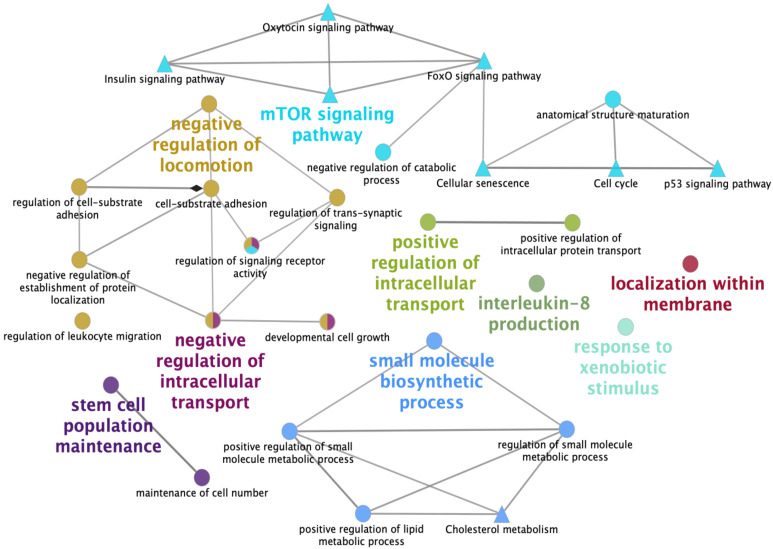
Schematic representation of biological processes and KEGG pathways found when comparing differentially expressed genes in embryos derived from sows infused with seminal plasma, relative to control embryos (BTS-group). The analysis of overrepresented functional categories was performed using the Cytoscape v3.0.0 application ClueGo v2.0.3. The following databases were used: GO subgroups in biological process (circles) and KEGG pathways (triangles). Terms are functionally grouped based on shared genes (kappa score), and are shown in different colors. The size of the nodes indicates the degree of significance, where the biggest nodes correspond to highest significance. The most significant term defines the name of the group. The following ClueGo parameters were used: biological process database (BP; date: 28.03.2019); GO three levels, 2–5 (first level = 0); minimum number of genes, 2; minimum percentage of genes, 2; GO term fusion; GO term connection restriction (kappa score), 0.4; GO term grouping, initial group size of 2 and 50% for group merge. The resulting network representation was manually adapted after removing unnecessary terms.

Pathway enrichment in the up-DEG list included pathways related to signal transduction (e.g., Apelin signaling, FoxO signaling and mTOR signaling), cellular processes (e.g., cell cycle, p53 signaling, cellular senescence, adhesion function and signaling pathways regulating pluripotency of stem cells) and the endocrine system (e.g., insulin signaling, progesterone-mediated oocyte maturation and relaxin signaling). These pathways contained genes with potential roles in key reproductive events, such as ApoA-I, CDK1, MAPK1, SMAD2, PRKAA1 and RICTOR (Table 2), that were highly expressed in the SP group compared to the BTS group. Two pathways, mineral absorption and p53-signaling, were enriched in both the down-DEG and the all-DEG lists and included genes, such as MT-2B, CDK2 and SERPINE1, with no apparent relationship to reproductive processes (Table 3). A network of the main biological processes and pathways found when comparing DEGs in embryos derived from SP-sows and BTS-sows is presented in Figure 3.

### 2.3. Validation of the Microarray Data

**Figure 4:**
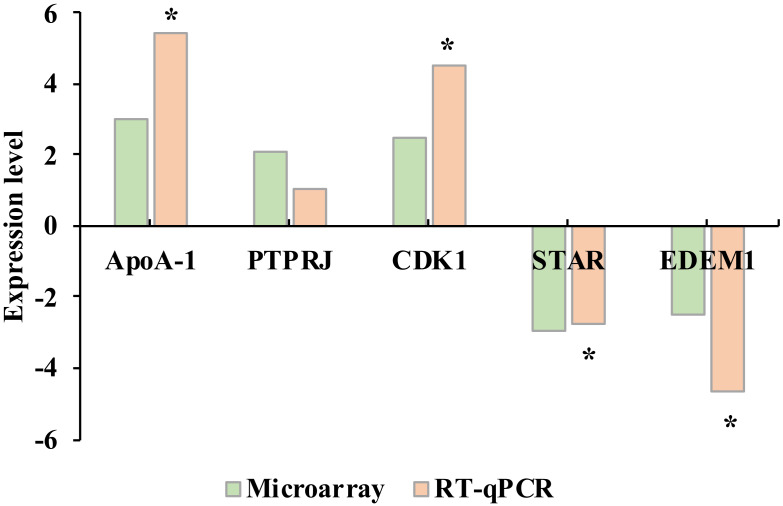
Validation of microarray results by real time quantitative PCR (RT-qPCR). The *γ*-axis represents the fold change between the seminal plasma and BTS groups. Asterisk indicates significant differences between seminal plasma and BTS groups by RT-qPCR analysis.

We selected five genes to verify the microarray results by real time quantitative PCR (RT-qPCR). These genes presented similar patterns of fold change under both methods (Figure 4), proving the microarray results were reliable.

## 3. Discussion

The results of this study show, to the best of our knowledge for the first time, that SP infusions during estrus modify the gene expression pattern of Day 6 pig blastocysts, upregulating the expression of genes with potential roles in embryonic development, implantation, or progression of pregnancy. These findings suggest that application of SP in ET-protocols could increase ET-outcomes by increasing the number of embryos succeeding during the crucial implantation window. Such a statement is based on the confirmation of previous findings [18,19,21], indicating that the effects of SP remain influential over time. Particularly, SP infusions at estrus advance embryonic development, and alters the expression of the endometrial genes and pathways potentially involved in embryonic development and maternal immune system tolerance during early pregnancy [19]. The present study showed that embryos derived from sows infused with SP at estrus had >200 DEGs than controls; many of these genes related to cellular, metabolic, biological and developmental processes. Interestingly, when we examined only the downregulated list of DEGs, only three pathways were enriched. Genes in these pathways (*ADORA1*, *CDK2 MT-2B*, *PTGS1* and *SERPINE1*) were neither related to embryo development nor implantation process. In contrast, certain genes with potential functions on embryo development, implantation and pregnancy were upregulated in SP-embryos compared to controls, and the corresponding enriched pathways were associated with cellular processes, endocrine system and signal transduction. Among these genes, *ApoA-I*, *CDK1*, *MAPK1*, *SMAD2*, *PRKAA1* and *RICTOR* have been reported as closely involved in different reproductive processes.

The *ApoA-I* gene encodes the major high-density lipoprotein (HDL) constituent, protein ApoA-I, which improves glucose metabolism and restricts inflammatory processes during pregnancy in several species [22]. A downregulation of the expression of this HDL has been associated with an increased risk of pre-eclampsia in humans [23], and its dysfunction enhances embryo toxicity by increasing cell susceptibility to protein degradation and structural modifications [24]. Physiologically, this gene was found upregulated in the endometrium of rodents during the implantation period [25], and it seems to promote several mechanisms to achieve a successful implantation process [26]. Moreover, an overexpression of the protein ApoA-I has been found in human early embryos and cultured blastocysts deemed of superior quality [27], which reinforces the idea that this protein is closely related with embryonic quality. In the present study, we found that the expression of *ApoA-I* gene was >3-fold higher in embryos from SP-sows than in controls, suggesting that the SP-treatment prior AI may enhance embryo survival and promote developmental processes by the activation of ApoA-I gene during pre-implantation. 

Several genes related to embryonic development were found upregulated in SP-treatment compared to the control group such as *CDK1*, *MAPK1*, *SMAD2*. *CDK1* has been reported to be fundamental for promoting both oocyte meiosis resumption [28] and mitotic cell divisions [29,30]. *MAPK1* is important for both embryonic and placental development [31], and insufficient expression of MAPK1 proteins decreases the adequate signal for trophoblast proliferation and invasion, due to their implication in cell proliferation, differentiation and development [32,33]. The *SMAD2* gene, which moderates the signal of the critical transforming growth factor (TGF)-ß pathway that regulates epiblast and germ layer development [34], was also upregulated in the SP-blastocysts. Interestingly, the TGF-ß pathway promotes the proliferation of immune-suppressive Regulatory T (Treg) cells, crucial to avoid embryo immune rejection [35,36]. In our study, SP-blastocysts evidenced an overexpression of that pathway, which is of great practical importance since SP infusions at estrus, could induce signaling pathways to further decrease the immune response of recipients to allogeneic embryos and, therefore, reduce the high embryonic loss, characteristic of current pig ET programs.

Another remarkably upregulated gene in SP-blastocysts was *PRKAA1*. This gene encodes the catalytic subunit of the 5’-prime-AMP-activated protein kinase (AMPK), a sensor of cell energy and metabolism. These sensor systems are necessary from the beginning of embryo development [37] since AMPK promotes cell polarity and cell cycle progression [38,39]. 

Another upregulated gene in SP-blastocysts with important functions in embryonic growth and development was *RICTOR*. The protein encoded by this gene plays a fundamental role in developing embryonic and extraembryonic tissues, as *RICTOR* deficiency led to retarded embryo development and lethality in mice [40,41]. Altogether, these findings could be associated with the advancement of development found in our previous studies, among SP-treated pig embryos [19]. 

Additionally, we observed a modification in the expression of some genes related to embryo organogenesis. For instance, *ATRX*, the alpha-thalassemia mental retardation X-linked gene, which was upregulated in SP-embryos, is transmitted to the early zygote through the maternal germ line and it has been associated with correct lamination of the neuro-progenitor cells in the brain of mice embryos [42]. Moreover, mutations of this gene triggered extensive early embryo death in mice [43]. Additionally, *DLX2* was overexpressed in SP-embryos. *DLX2* belongs to the homeobox gene family, which is widely studied for its role in segmentation and cellular differentiation [44,45]. This particular gene has been previously described in the embryonic neural tube, and is known to participate in the segmentation of the embryonic forebrain [46]. 

In conclusion, the effects of SP infusions during estrus seem long-lasting enough to influence development and the transcriptional expression profile of preimplantation Day 6 pig blastocysts. The SP induces genes and pathways associated with embryo development, implantation and maternal immune tolerance to hemi-allogeneic embryos to be particularly affected. The potential application of SP infusions, in both donor and recipient, of highest interest for the pig industry, need to be established.

## 4. Materials and Methods 

### 4.1. Ethics Statement

The procedures used in this work were carried out complying with the directive 2010/63/EU and were evaluated and approved by the Ethical Committee for Experimentation with Animals of the University of Murcia, Murcia, Spain (research code: 22072015), and by the Murcia Autonomous Government (01062016/125089).

### 4.2. Animals

Weaned crossbred (Landrace × Large-White) sows (2–6 parity), with similar lactation periods (21 to 24 days), were used for the experiment. After weaning, the sows were housed in single crates in a barn with automatic ventilation at a commercial farm (Porcisan SA, Murcia, Spain). Mature boars (1 to 4 years of age) allocated in individual pens in the same farm and undergoing regular semen collection for artificial insemination (AI), were used as semen donors. All animals had access to water ad libitum and were fed commercial diets conforming to their nutritional needs.

### 4.3. Detection of Estrus

The detection of estrus was carried out by a skilled technician by allowing snout-to-snout contact of females with vasectomized mature boars, and by applying back pressure, twice a day, starting on the first day after weaning. Sows exhibiting standing estrus reflex in the presence of the boar were considered to be in estrus. The day of the onset of estrus was termed as Day 0.

### 4.4. Estrus Synchronization and Superovulation

Weaning was used to synchronize estrus among sows. Superovulation was induced with equine chorionic gonadotropin (eCG; Folligon, Intervet, Boxmeer, The Netherlands) and human chorionic gonadotropin (hCG; Veterin Corion, Divasa, Farmavic S.A., Barcelona, Spain), as previously described [47]. Briefly, a dose of 1000 IU eCG was administrated (im) at 24 h post-weaning. Sows in estrus at 72–96 h post-eCG were treated (im) with 750 IU hCG at the onset of estrus.

### 4.5. Artificial Insemination

The sperm-rich fraction of the ejaculate was extended in BTS and conserved at 18 °C for a maximum of 24 h. Post-cervical AIs were performed at 0 and 24 h after the onset of estrus with 40 mL sperm doses containing 1.5 × 10^9^ spermatozoa.

### 4.6. Seminal Plasma Preparation 

Heterologous SP was obtained from gel-free ejaculates collected from 8 healthy boars of proven fertility, as previously described [19]. Briefly, after collection, the ejaculates were immediately transported to the laboratory (within 2 h) and centrifuged three times at 1500× *g* at 17 °C for 10 min. The last supernatant was in all cases free of sperm. The SP-supernatants from the 8 boars were pooled, separated into aliquots of 40 mL in AI bottles and stored at −20 °C until use. The SP doses were thawed at 37 °C for 20 min immediately before inseminations and infused through a post-cervical catheter into the uterus.

### 4.7. Embryo Collection and Evaluation

Embryos were surgically obtained on Day 6 of the cycle. Sows were sedated with 2 mg/kg of body weight (im) of azaperone (Stresnil^®^, Sanochemia Pharmazeutika AG, Neufeld/Leitha, Austria). General anesthesia was induced with sodium thiopental (7 mg/kg of body weight, intravenous; B.Braun VetCare SA, Barcelona, Spain) and maintained with 3–5% isoflurane in air (IsoFlo^®^, Madrid, Spain). After exposure of the genital tract, the embryos were collected by flushing the tip of each uterine horn with 30 mL of washing medium at 37 °C (Tyrode’s lactate-HEPES-polyvinyl alcohol medium [48]) as previously reported [19]. 

Recovered embryos were evaluated to assess their quality and stage of development following the morphological criteria of the International Embryo Transfer Society [49]. Embryos with a differentiated blastocoel, inner mass cell and trophoblast and increased diameter and very thinned zona pellucida were classified as expanded blastocyst. Only expanded blastocysts deemed of excellent morphology were used in the experiment.

### 4.8. Total RNA Extraction

Total RNA was isolated from samples using the RNeasy Micro kit (P/N 74004; Qiagen Iberica, Madrid, Spain), according to the manufacturer’s instructions. The concentration and quality of the isolated RNA was assessed with the Bioanalyzer 2100 (Agilent, Santa Clara, CA, USA). All extracted samples had an acceptable quality, with RNA integrity number values greater than 8.

### 4.9. Microarray Hybridization

The porcine genome microarrays were purchased from Affymetrix (Santa Clara, CA, USA), and the different steps of the procedure were accomplished according to the instructions of the manufacturer. An amount of 650 pg of total RNA from each sample was transformed to ss-cDNA using the GeneChip 3’ IVT Pico Reagent kit (P/N 902790; Affymetrix, ThermoFisher Scientific, Madrid Spain). The quantity and quality of cDNA were measured using Nanodrop 2000 (ThermoFisher Scientific) and Bioanalyzer 2100 (Agilent, Santa Clara, CA, USA), respectively; cDNA targets were cleaned up and after fragmentation and terminal labelling, 4.5 µg of fragmented and biotinylated cDNA were loaded on the array chip (Porcine gene 1.0 ST GeneChip^®^ Cartridge Array, Affymetrix), and hybridized during incubation at 45 °C under rotation at 60 revolutions/min for 16 h. The hybridized array chip was then unloaded, subjected to washing and staining using the GeneChip Hybridization, Wash and Stain kit (P/N 90720; Affymetrix), and finally scanned using the Affymetrix GeneChip^®^ scanner GCS3000. The Affymetrix GeneChip^®^ Porcine Genome Array contains 23,256 transcripts from 20,201 genes that provide a comprehensive coverage of the *Sus scrofa* transcriptome. After scanning, microarrays data were processed using Affymetrix Expression Command Console (Affymetrix) and all samples overcame the quality criteria. 

### 4.10. Analysis of the Microarray Data

Data were first normalized using the Robust Multichip Average (RMA) method [50]. Raw intensity values were background corrected, log2 transformed and then quantile normalized, in order to obtain an individual intensity value for each probe set. The statistical analysis was performed using software of the Partek Genomics Suite & Partek Pathways (Partek Incorporated, St. Louis, MO, USA). The PCA was used to describe variations in the transcriptome between samples and to provide the overall structure of the analyzed dataset. A single-factor ANOVA was used for detecting DEGs between SP-embryos and BTS-embryos. Genes were considered differentially expressed if the un-adjusted *p*-value of the ANOVA was lower than 0.05 and the absolute fold change was 2, between SP and BTS groups. 

The enrichment analysis of the Gen Ontology (GO) terms was performed with Partek to detect overrepresented biological processes, cellular components and molecular functions, using Fisher’s exact test. This gene enrichment analysis evaluates if genes within a specific class differs by function. Pathway analysis was performed with Partek pathway function using Fisher’s exact test. The *p*-value is calculated using the cumulative hypergeometric distribution test [51]. The analysis of the GO terms and pathways performed with Partek is based on the KEGG database [52]. Gene networks were performed using ClueGO plug-in of Cytoscape software [53].

### 4.11. RT-qPC Assay

Microarray results were validated by RT-qPCR of 5 randomly selected DEGs, 3 upregulated (*ApoA-I*, *PTPRJ* and *CDK1*) and 2 downregulated (*STAR* and *EDEM1*). The RNA samples used for the RT-qPCR assay were the same samples used for the microarray analysis. Total RNA was reverse transcribed into the first-strand cDNA using the Maxima H Minus First Strand cDNA Synthesis Kit (ThermoFisher Scientific). Incubations were performed during 10 min at 25 °C followed by 15 min at 50 °C. The reactions were terminated at 85 °C for 5 min. The primers (Table 4) were designed using the Primer Express^™^ software v3.0.1 (Applied Biosystems, Foster City, CA, USA), and were commercially synthesized. The RT-qPCR analysis was performed with 1-μL iTaqTM Universal SYBR Green Supermix (Bio-Rad Laboratories, Hercules, CA, USA), 500 nM for each forward and reverse primer and 3 ng of total DNA in 10- μL total volume. The reactions were performed in a QuantStudio™ 5 Real-Time PCR System (Applied Biosystems). The cycling profile was 2 min at 50 °C for Uracil-DNA glycosylase activation, 10 min at 95 °C for a previous denaturation, followed by 40 cycles of 15 s at 95 °C 1 min at 60 °C. The reaction specificity was monitored through a melt curve analysis (included in each PCR reaction). Each sample for each gen was run in triplicate. The Pfaffl method [54] was used to identify the relative expression of each gene, which was based on the gene efficiency (E) and the mean Ct value of the gene in a sample from SP group versus the control sample, and normalized to the housekeeping reference gene *peptidylprolyl isomerase A* (*PPIA*). The corresponding gene efficiency was calculated according to the equation: E = 10^(−1/slope)^. The RT-qPCR data were analyzed by Student’s *t*-test using the IBM SPSS 24.0 Statistics package (IBM, Chicago, IL, USA). A *p*-value of < 0.05 was considered statistically significant.

### 4.12. Experimental Design

**Figure 5:**
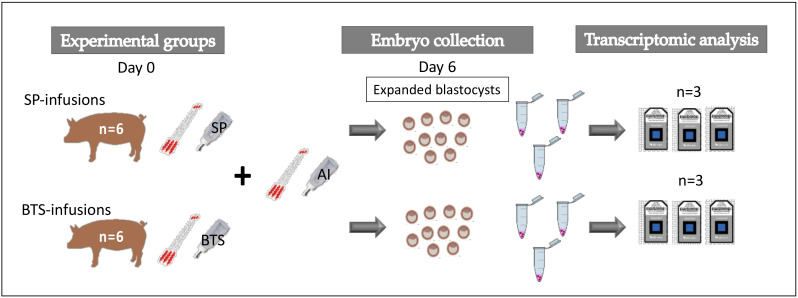
Schematic representation of the experimental design. Experimental groups: Sows infused with seminal plasma (SP) or BTS extender (Control) 30 min before each of two artificial inseminations (AI). Day 6 blastocysts were collected and pooled within each group. Pooled embryos (*n* = 10/tube) were subjected to transcriptomic analysis (microarrays).

To evaluate the effects of additional heterologous SP-infusions on the embryo transcriptome, a total of 12 postweaning estrus sows received 40-mL intrauterine infusions of either heterologous, pooled SP (*N* = 6) or BTS (control group; *N* = 6) 30 min before each of two AIs (Figure 5). The experiment was conducted in a total of 2 replicates and, within each replicate, all sows from the two groups were inseminated with sperm doses from the same boar. At Day 6 of the cycle, sows were subjected to laparotomies to collect the embryos. Only embryos at the expanded blastocyst stages were used for transcriptome analysis. A total of 30 embryos collected from the 6 sows of each group (SP or BTS), and deemed of excellent morphology, were pooled together. Pooled embryos were distributed into 3 tubes within each group containing 10 embryos per tube and 5 µL of PBS. Samples were immediately snap-frozen in liquid nitrogen and stored at −80 °C until further use. A total of 6 microarrays were performed (3 microarrays for SP-treated embryos and 3 for BTS-control embryos). Microarray results were verified by RT-qPCR. For this validation, 3 biological replicates (3 samples per group), and 3 technical replicates per sample were performed.

## Figures and Tables

**Table 1:** Enrichment analysis of Kyoto Encyclopedia of Genes and Genomes (KEGG) pathways. Pathways depicting significant enrichment *p*-value of gene sets in all-Differentially Expressed Genes (DEG), up-DEG and down-DEG target lists. Non-significant values are displayed in grey shadow.

Pathway ID	Pathway Name	Enrichment *p*-Value		
		All	Up	Down
ssc04218	Cellular senescence	0.002	0.010	0.156
ssc04115	p53 signaling	0.002	0.045	0.035
ssc04371	Apelin signaling	0.006	0.030	0.124
ssc04979	Cholesterol metabolism	0.008	0.036	0.194
ssc04110	Cell cycle	0.021	0.030	0.428
ssc04520	Adherents junctions	0.022	0.005	-
ssc04140	Autophagy-animal	0.025	0.036	0.446
ssc04068	FoxO signaling	0.027	0.036	0.452
ssc04914	Progesterone-mediated oocyte maturation	0.041	0.061	0.333
ssc04978	Mineral absorption	0.045	-	0.013
ssc04923	Regulation of lipolysis in adipocytes	0.080	-	0.025
ssc04926	Relaxin signaling	0.103	0.020	-
ssc04550	Signaling pathways regulating pluripotency of stem cell	0.112	0.021	-
ssc04910	Insulin signaling	0.114	0.037	-
ssc04150	mTOR signaling	0.141	0.043	-

**Table 2:** Over-expressed (*p* < 0.05) biological pathways examined with KEGG database in blastocysts collected six days after seminal plasma infusions prior to insemination relative to control blastocysts.

Pathway ID	Pathway Name	Enrichment Score	Genes Altered (%)	Gene List
SSC04520	Adherents junctions	5.3	6.7	*PTRJ, MAPK1, SMAD2*
SSC04218	Cellular senescence	4.6	3.6	*MAPK1, SMAD2, CDK1*
SSC04926	Relaxin signaling	3.9	6.9	*MAPK1, Smad2, COL4A1*
SSC04550	Signaling pathways regulating pluripotency of stem cell	3.9	3.9	*Smad2, MAPK1, Hesx1*
SSC04110	Cell cycle	3.5	3.4	*Smad2, Stag1, CDK1*
SSC04371	Apelin signaling	3.5	3.4	*PRKAA1, SMAD2, MAPK1*
SSC04979	Cholesterol metabolism	3.3	5.1	*ApoA-I*
SSC04068	FoxO signaling	3.3	3.1	*PRKAA1, SMAD2, MAPK1*
SSC04140	Autophagy–animal	3.3	3.1	*PRKAA1, ATG4C, MAPK1*
SSC04910	Insulin signaling	3.3	3.1	*PRKAA1, GYS1, MAPK1*
SSC04150	mTOR signaling	3.1	2.9	*MAPK1, PRKAA1, RICTOR*
SSC04115	P53 signaling	3.1	4.5	*CDK1, IGF-BP3*

**Table 3:** Under-expressed (*p* < 0.05) biological pathways examined with KEGG database in blastocysts collected six days after seminal plasma infusions prior to insemination, relative to control blastocysts.

Pathway ID	Pathway Name	Enrichment Score	Genes Altered (%)	Gene List
SSC04978	Mineral absorption	3.6	6.7	*MT-2B*
SSC04923	Regulation of lipolysis in adipocytes	3.3	5.7	*PTGS1, ADORA1*
SSC04115	P53 signaling	3.3	3.1	*CDK2, SERPINE1*

**Table 4:** Information on primer sequences used for real time quantitative PCR and amplicon sizes.

Gene	Accession Number	Primers (5′–3′)	Size (pb)	Efficiency (%)	R^2^
*ApoA-1*	NM_214398.1	F: CGATCAAAGACAGTGGCAGA	166	75.5	0.96
		R: TCCAGGTTGTCCCAGAACTC			
*PTPRJ*	XM_013994325.1	F: CCAGCAAGACAACGCAGATA	179	96.6	0.99
		R: GTGGAAGGAGGCTACAGGTG			
*CDK1*	NM_001159304.2	F: AGGCTAGAAAGTGAAGAGGAAGG	193	115.0	0.99
		R: TGAACTGACCAGGAGGGATAG			
*STAR*	NM_213755.2	F: CCCCGAGACTTTGTGAGTGT	186	151.9	0.99
		R: CAGCCAGGTGAGTTTGGTCT			
*EDEM1*	XM_003483209.3	F: AGGACCAAGTGGAAAAGTCTG	160	101.6	1.00
		R: CAAATCAAGCCAACCATCTG			
*PPIA*	XM_021078519.1	F: CTGAAGCATACGGGTCCTGG	100	98.1	1.00
		R: CCAACCACTCAGTCTTGGCA			

## Supplementary Materials

Supplementary Materials can be found at https://www.mdpi.com/1422-0067/21/10/3662/s1.
